# Child Caregiver’s healthcare seeking behavior and its determinants for common childhood illnesses in Addis Ababa, Ethiopia: a community-based study

**DOI:** 10.1186/s13052-021-01049-w

**Published:** 2021-04-21

**Authors:** Martha Bellete, Moges Muluneh Boke, Melaku Kindie Yenit

**Affiliations:** 1 Federal Ministry of Health, Nifas Slik lafto Health office, Addis Ababa, Ethiopia; 2 Department of Reproductive Health, Institute of Public Health, College of Medicine and Health Science, University of Gondar, Gondar, Ethiopia; 3 Department of Epidemiology and Biostatistics, Institute of Public Health, College of Medicine and Health Science, University of Gondar, Gondar, Ethiopia

**Keywords:** Childhood illnesses, Health care-seeking, Caregiver

## Abstract

**Background:**

Appropriate healthcare-seeking behavior and access to the health care facility is key to improving health service utilization. Although the accessibility of comprehensive childhood disease intervention services in Ethiopia has been modified at the community level, the use of such health care services has remained limited. Therefore, this study aimed to assess the healthcare-seeking behavior of common childhood illness and its determinants.

**Methods:**

A community-based cross-sectional study design was used. A multi-stage sampling method was used to recruit eight hundred and thirty-four study participants. A pre-tested and standardized questionnaire was used to collect data. The collected data were visually checked for incompleteness and entered into the statistical software Epi-info version 7 and exported to SPSS version 20 software for descriptive and bi-variable analysis. To identify variables associated with the healthcare-seeking behavior. Logistic regression analysis was performed. Adjusted odds ratios with a 95% confidence interval were used to see the strength of association, and variables with *P*-values of < 0.05 were considered statistically significant.

**Results:**

The proportion of health care seeking behavior of care-givers for childhood illness was 69.5% (95% CI, 66.4, 72.4%). The education level of caregiver (AOR: 1.61, 95% CI: 1.01–2.60), knowledge of childhood illness (AOR: 2.02, 95% CI: 1.46–2.79), cough (AOR: 1.94, 95% CI: 1.39–2.71) and diarrhea (AOR: 2.09, 95% CI: 1.46–2.99) as main symptoms of illness and perceived severity of illness (AOR:3.12, 95% CI: 2.22–4.40) were significantly associated with healthcare-seeking behaviors of caregivers.

**Conclusion:**

Low healthcare-seeking behavior was observed for childhood illnesses. Educational level, knowledge of childhood illness, cough, and diarrhea as primary symptoms of illness, and perceived severity of caregiver illness were significant associated with healthcare-seeking behavior. Therefore, interventions that strengthen the caregiver’s awareness of childhood illness and danger signs need to be considered. Besides, addressing the identified associated variables to healthcare-seeking behavior is critically important to curb the problem.

## Background

Children are at the center of global efforts to improve the well-being of human beings. Over the past three decades, the rate of under-five child mortality failed dramatically from 93 deaths per 1000 live birth in 1991 to 39 deaths in 2019 per 1000 live births. Despite substantial progress in reducing child mortality worldwide over the past three decades, more than five million children die each year from the most preventable causes, and the issue of child mortality remains persistent [1]. In sub-Saharan Africa, 76 children die in every 1000 live births, while in developed nations less than eight child deaths occur in every 1000 live births [2].

In the last three decades, Ethiopia has made progress in reducing under-five child mortality. In 2019, the under-five child mortality rate dropped from 200 deaths per 1000 live births to 51 deaths per 1000 live births, with an annual reduction rate of 4.7% [2]. Despite this surprising success over the past three decades, children under the age of five in Ethiopia still die from preventable or treatable conditions every year. A significant proportion of deaths and illnesses due to these diseases can be avoided by the low cost and available preventive interventions [3].

For each of the major killers of children under five; pneumonia, newborn problems, diarrhea, and malaria; there are several high impacts, low-cost preventive and curative interventions that have been undertaken to save the majority of lives of Ethiopian under-five children. This includes the introduction of a standard protocol for Integrated Management of Neonatal and Children Illnesses (IMNCI) and the Community-based Case Management of Common Childhood Illnesses, part of the HEP initiative in 2010, it could prevent many deaths among children if they are presented for appropriate and timely care [4]. The IMNCI strategy, besides improving providers’ skills in managing childhood illness also aims to improve families’ care-seeking behavior [5, 6]. Ethiopia despite expansion and implement integrated management of childhood illness strategies, the health care use for childhood illness is still low [7, 8].

Improving the care-seeking behavior of families could significantly contribute to reducing child mortality in developing countries, and most child deaths result from delays in seeking adequate care and not seeking any care. According to the 2016 Ethiopian Demographic and Health Survey (EDHS) report, just 43, 35, and 30% of children with diarrhea, fever, and ARI, respectively, were taken to a health facility for seeking health care service [9]. Such a study found that caregivers’ healthcare-seeking behavior for these common childhood illnesses remains poor. This research, therefore, aimed to estimate the level of healthcare-seeking behavior and identify factors affecting the health care seeking behavior of caregivers to find strategies to improving child health outcomes.

## Methods

### Study design and period

A community-based cross-sectional study was employed from January to February 2020.

### Study setting

This study was conducted from January to February 2020 in the Nifas Silk Lafto sub-city, Addis Ababa. The estimated area of Addis Ababa city is 174.4 km^2^ and has an estimated density of 5535.8 people per square kilometer [10]. The health service area coverage of the city is 90%. The rate of health service use is 1.71 per capita per year [11]. There are 13 districts, 465,428 population, 2 private hospitals, and 10 health facilities in the Nifas Silk Lafto Sub-city.

### Population

The study population was mothers with under-five children who had an illness during the previous 2 weeks of data collection. In the absence of a mother, another child caregiver in the household was interviewed to reduce the non-response rate.

### Sample size determination

To estimate the required sample size, single proportion population formula was used with the following assumption, 30% proportion of health care seeking to the ill child [12], 5% level of significance, 95% confidence interval (Z α/2 = 1.96), 5% absolute precision or margin of error, 2 design effect and 10% non-response rate. Finally, the sample size required for the study was 875.

### Sampling methods

A multi-stage sampling technique has been employed to recruit study participants. Six woredas were selected by a random sampling method from a thirteen woreda (the smallest administrative area in the city that contains five to six sub-districts or Ketena). The total sample size is proportionally allocated to each woreda. To get study participants from selected woreda cluster methods were employed. Three Ketenas were considered to take mothers with sick children. The clusters were known to be Ketenas (the smallest administrative unit in the Woreda) with a population of at least 4000, although often it depends on the geographical location. All mothers with under-five children who were ill in the previous 2 weeks were interviewed until the sample size per woreda was obtained.

### Operational definition

#### Healthcare-seeking behavior

defined as caregiver’s response for signs and symptoms of illnesses to reduce the severity, complication, or even death after recognized child’s illness and if caregiver reported visiting any health institutions considered as having healthcare-seeking behavior.

#### Fever

If mother perceived as fever or hot body for any child 2 weeks preceding the survey.

#### Diarrhea

If the caregivers described their sick children had three or more loose or watery stools per day at any time within the 2 weeks before the survey.

#### Cough

all cases who had cough less than 2 weeks preceding the survey as perceived by mothers or caretakers.

#### Caregiver

is a mother or equivalent family member who is primarily responsible for caring for a child and who could explain enough about the sickness-related behaviors of a child.

#### Common childhood illness

common childhood illness includes diarrheal, fever, and cough.

#### Knowledge score

respondents who scored above or equal to the mean were labeled as having optimal knowledge and those who scored below the mean were labeled as having poor knowledge about childhood illnesses [13].

### Data collection tool and procedure

Pre-tested and standardized questionnaires were used to interview child caregivers. The questionnaires were developed through reviewing different literature [14–16]. To check its consistency, the questionnaire was initially prepared in English and later translated into the local language (Amharic) and back to English. The questionnaire contains three-section, socio-demographic characteristics, child’s illness-related characteristics, and healthcare-seeking behavior of caregiver. Six diploma nurse data collectors were recruited and trained for 2 days on the objectives of the study, and the approaches to data collection. Field supervision and follow-up were made by the principal investigator and two Bsc nurses to monitor the progress and quality of the data collection process.

To ensure data quality, different methods of data quality assurance were employed. Two days of intensive training were given to data collectors and supervisors. A pre-test was performed in a 5% of sample size in Arada- sub city woreda 01. The collected data was checked daily base on the field to avoid incompleteness and inconsistency by principal investigators and supervisors.

### Data management and analysis

Each questionnaire was manually checked, coded, and entered into EPI info version 7 statistical software and exported to a statistical package of social science (SPSS) Version 20.0 software for analysis. Univariable, bivariable and multivariable analyses were performed. Percentage, frequency, and mean were calculated for the socio-demographic characteristics of caregivers. Finally, binary logistic regression models were fitted as a multivariable analysis tool to obtain odds ratios, 95 percent confidence intervals, and control confounders. Variables with less than 0.05 *p*-values in the multivariable logistic regression analysis with a 95% confidence interval were considered significant. The analysis results were presented using tables, figures, and text as appropriate.

## Results

### Socio-demographic characteristics of respondents

A total of 834 care-givers had under-five children with common childhood illness in the past 2 weeks before the study was interviewed. The minimum and maximum ages of respondents were 18 and 70 years respectively. The mean (SD) age of respondents was 30 (+ 6.7) years. Most 798 (95.7%) of respondents were mothers as primary caregivers, 768 (92.1%) of them were currently in marriage. Over one-fifth 189 (22.7%) of caregivers had no formal education. More than one-third, 323 (38.7%)) had poor knowledge of childhood illness. More than half 483 (57.9%) of the respondents were Orthodox Christian religion followers. The mean (SD) number of living children and under-five children a caregiver was 2.0 (+ 1.3) and 1.5 (+ 0.6), respectively. The average time to reach the nearest health facility was 18 min. (Table 1).

**Table 1:** Socio-demographic characteristics of the respondents at Nifasilk Lafto sub-city, Addis Ababa; January 2020

variables (***N*** = 834)	Number	Percent (%)
Age of respondents (In years)		
18–25	200	24.0
26–34	453	54.3
35–43	151	18.1
> 44	30	3.6
Relationship to the sick child		
Mother	798	95.7
Others ^a^	36	4.3
Educational Level		
No formal education	189	22.7
Grade 1–4	229	27.5
Grade 5–8	227	27.2
Grade 9 and above	189	22.7
Knowledge of childhood illness		
Poor	323	38.7
Optimal	511	61.3
Current marital status		
Not currently married	66	7.9
Currently married	768	92.1
Religion		
Orthodox	483	58.1
Muslim	202	24.3
Other^b^	147	18.6
Number of live children		
One	325	39.0
Two and above	509	61.0
Number of under 5 children		
One	469	56.2
Two and above	365	43.8
Distance to HC (in time)		
Less than10 Min	246	31.5
11–20 Min	278	35.5
20 Min and above	258	33.0

^a^Others includes grandmother, father, and sister ^b^ includes protestant and catholic

### Common childhood illness-related characteristics

Nearly half 390 (46.8%) of children with common childhood illnesses were females and their median age was 25.1 months. The three most common reported symptoms were fever 724 (86.8%), cough 554 (66.4%), and diarrhea 298 (35.7%). More than two-thirds (71%) of children with common childhood illnesses had more than one symptom of illness. Almost three-quarters 608 (72.9%) of caregivers were perceived the recent childhood illness as severe. Just over half 423 (52.3%) of caregivers perceived the severity of the illness through the symptoms noticed in their child. (Table 2).

**Table 2:** Common childhood illness-related characteristics of under-five children in Nifas silk lafto sub-city, Addis Ababa; January 2020

Variables (***N*** = 834)	Number	Percent (%)
Sex of sick child		
Female	390	46.8
Male	444	53.2
Age of sick child		
Less than 24 months	401	48.1
24 months and above	433	51.9
Main symptoms		
Cough	554	66.4
Diarrhea	298	35.7
Fever	724	86.8
Perceived severity		
Sever	608	72.9
Not sever	223	26.7
Difficult to judge	3	0.4
Reasons for the perceived severity		
Had more than one symptom	216	26.7
Learned that the symptom indicates the seriousness of the child illness	423	52.3
From experience, assuming it resolve by itself	177	21.9
Fear of child dying of the illness	40	4.9

### Healthcare seeking behavior for childhood illness

In this study, the magnitude of healthcare-seeking behavior of caregivers for common childhood illnesses was 69.5% (95% CI: 66.4, 72.4). Health centers and private clinics were the most preferred health facilities to seek treatment for their children among caregivers with 373 (64.3%) and 198(34.1%), respectively. Of health facility visitors, 304 (52.4%) respondents took their child to health facilities after the second days of the onset of illness and only 38 (6.6%) took immediately within 24 h from the recognition of illness, and 306 (52.8%) reported that the reason for preferring the health facility was “seeking better treatment of the child illness”. Care was more commonly sought at a health facility for multiple symptoms presentation than a single symptom.

Nearly a third 254 (30.4%) of caregivers were not sought care from health facilities for their child’s illness; 40(4.9%) purchased medicine from drug shops, and 89 (10.7%) gave home treatment with traditional remedies. No care at all for 70 (8.4%) of children. The main reasons for seeking health care other than health facilities were the cost of treatment 198 (77.9%), perception of the illness as not serious 42 (16.5%). (Table 3).

**Table 3:** Healthcare seeking behavior of caregivers for childhood illness in Nifas silk lafto sub-city, Addis Ababa; January 2020

Variables	Number	Percent
Care sought for the child illness (*n* = 834)		
Health facility	580	69.5
Purchased medicine from the drug store	40	4.9
Taken to religious /traditional healer/	55	6.6
Given home treatment	89	10.7
Waited for illness to subside	70	8.4
Time of health facility visited (*n* = 580)		
Immediately	38	6.6
On the first day	83	14.3
On the second day	155	26.7
After the second day	304	52.4
Types of health facility visited (*n* = 580)		
Hospital	16	2.7
Health center	373	64.3
Private clinic	198	34.1
Reason for the preferred health facility (*n* = 580)		
Since the illness was sever	54	9.3
Fear of illness was worsening	41	7.0
Seeking better treatment	306	52.8
Facility was nearby	130	22.4
People advised	49	8.5
Reason for not visiting health facility (*n* = 254)		
Treatment expensive	198	77.9
The distance of the health facility	14	5.6
The illness was not serious	42	16.5

### Factors associated with healthcare-seeking behavior for common childhood illness

In the bivariable logistic regression analysis knowledge and perceived severity of illness of caregivers, age and main symptoms of illness of the sick child were significant associated with healthcare-seeking behavior at a *p*-value of less than 0.2.

In multiple logistic regression education levels, knowledge of childhood illness, perception of the severity of the illness, cough, and diarrhea childhood illness remained as predictors of health care seeking behaviors of the child caregivers. Caregivers were education level grade nine and above (AOR: 1.63, 95% CI: 1.01–2.64), knowledge of childhood illness (AOR: 2.04, 95% CI: 1.47–2.82), cough as the main symptom of illness (AOR: 1.94, 95% CI: 1.39–2.72), diarrhea as the main symptom of illness (AOR: 2.15, 95% CI: 1.50–3.09), and perceived severity of illness (AOR: 3.35, 95% CI: 2.36–4.74) were significantly associated with healthcare-seeking behaviors of caregivers. Divergently, fever as the main symptom of illness and age of the sick child were not significantly associated with the healthcare-seeking behavior of caregivers. (Table 4).

**Table 4:** Factors associated with healthcare-seeking behavior for common childhood illnesses in Nifassilk Lafto Sub-city, Addis Ababa; January 2020

Factors	Health facility visited for common childhood illnesses		COR 95%CI	AOR 95%CI
	No, N(%)	Yes, N(%)		
**Education level**				
No formal education	65(34.4)	124(65.6)	1.00	1.00
Grade 1–4	87(38.0)	142(62.0)	1.16 (1.01,1.28)	1.21 (1.08, 1.59) *
Grade 5–8	53(23.3)	174(76.7)	1.72(1.12, 2.65)	1.88 (1.18, 2.99) **
Grade 9+	49(25.9)	140(74.1)	1.50(1.19, 2.33)	1.63 (1.01, 2.64) *
**Knowledge of childhood illness**				
Poor	126(39.0)	197(61.0)	1.00	1.00
Optimal	128(25.0)	383(75.0)	1.91 (1.42, 2.58)	2.04 (1.47, 2.82) ***
**Cough as the main symptom**				
No	107(38.2)	173(61.8)	1.00	1.00
Yes	147(26.5)	408(73.5)	1.71 (1.26, 2.33)	1.94(1.39, 2.72) ***
**Diarrhea as the main symptom**				
No	193(36.0)	342(64.0)	1.00	1.00
Yes	61(20.5)	236(79.5)	2.19 (1.57, 3.05)	2.15 (1.50, 3.09) ***
**Fever as the main symptom**				
No	40(36.4)	70(63.6)	1.00	1.00
Yes	214(29.6)	510(70.4)	1.36 (0.90, 2.07)	1.07 (0.67, 1.71)
**Perceived severity of illness**				
Sever	141(23.2)	467(76.8)	3.31 (2.40, 4.57)	3.35 (2.36, 4.74) ***
Not sever	113(50.0)	113(50.0)	1.00	1.00
**Age of the sick child**				
Less than 24 months	111(27.3)	296(72.7)	1.34((1.00,1.81)	1.27(0.91, 1.75)
24 months and above	143(33.5)	284(66.5)	1.00	1.00

*Significant at *P* < 0.05, ** Significant at *P* < 0.01, *** Significant at *P* < 0.001

## Discussion

This study presented several insights about the healthcare-seeking behavior of caregivers for children with reported cough, diarrhea, and fever in the last 2 weeks, type of facilities visited for seeking care, socio-economic and demographic factors affecting healthcare-seeking behaviors of caregivers.

In this study, the level of healthcare-seeking behavior among caregivers of children with common childhood illnesses was 69.5% (95% CI: 66.4, 72.4). This finding was consistent with a study obtained from Southern Nations, Nationalities, and Peoples of Ethiopia (68.5%) [13]. However, this finding was lower than the studies obtained from Ethiopia Oromia (74.6%) [17] and Tigray (76.2%) [18] regions. The reason for this discrepancy might be the availability of community-level health care services in rural Ethiopia called integrated childhood case management (ICCM). In rural Ethiopia, health extension workers deliver integrate childhood case management (ICCM) at the community level. This integration of ICCM service with a health extension program enhances appropriate care-seeking behaviors for childhood illnesses in rural Ethiopia [15, 19]. On the other hand, the level of healthcare-seeking behavior of the current study is higher than studies obtained from Addis Ababa (56.6%) [20], Northwest of Ethiopia (48.8%) [15], and urban slums of Malawi (61%) [21]. The reason for this variation might be differences in the definition of sick children. In the current study, under-five children who had an illness within the previous 2 weeks of the data collection period were considered whereas, the studies obtained from Oromia and Malawi consider up to 1 month [21, 22].

According to the multivariable logistic regression analysis, the higher odds of health care seeking behavior were noticed among caregivers with higher education and knowledge, perceived severity of illness, and reported symptoms of cough and diarrhea. In the current study, caregivers who had formal education were more likely to visit health facilities for childhood illness than caregivers with no formal education. The finding was consistent with other studies conducted in Ethiopia [7, 15] Kenya, and sub-Saharan countries [22]. It is well known that education affects the healthcare-seeking behavior of the community. Therefore, educated mothers/caregivers can easily recognize the severity of diseases and seek healthcare.

This study found that caregivers who had optimal knowledge of childhood illnesses were more likely to seek care from health facilities than with poor knowledge. This finding is similar to other findings in Ethiopia [13] and Malawi [21]. The knowledge and perception of caregivers about childhood illnesses in children usually determine the way the illness was initially managed at home as well as their care-seeking behavior. It is well known that knowledge can be acquired through education and knowledgeable caregivers can easily understand the severity of illnesses and seek healthcare appropriately.

Most studies demonstrated that caregivers’ responses and practices were frequently influenced by the types of illness the child had and on their perception of the severity of illness [7, 15]. In this study, we found that cough and diarrhea as the main symptoms of child illness had a significant association with healthcare-seeking behavior. The odds ratio shows that children with cough and diarrhea were more likely to visit health facilities than no cough or diarrhea, which finding is consistent with reports of other studies in Ethiopia [10, 13] and Malawi [21]. It is known that having a childhood illness is the main pushing factor to visit a health facility. However, in this study, the fever of a child no role in the healthcare-seeking behavior of the caregiver. This finding contradicts some other studies finding obtained from Ethiopia [7]. This difference is may be due to seasonal and geographic differences in study areas [23, 24].

In this study, the higher odds of healthcare-seeking behavior were reported among caregivers who perceived the severity of child illness. This finding is similar to the studies in Ethiopia [17], Nigeria [8], and Equatorial Guinea [25]. This is because the perceived severity of illness is a good indication of the decision-making process. When caregivers acknowledge the severity of illness, they were more likely to seek health care.

In this study, the age of the sick child did not show any association with the healthcare-seeking behavior of caregivers, even though caregivers seek more care for younger children. This finding contrast to other studies finding from Ethiopia [26].

### Strength and limitation of the study

Limitations are acknowledged for this study. The fever, and cough illness data were assessed based on caregivers’ perception of illness without proof by health care personnel, which could introduce bias in the study. Moreover, this study relied on self-reported information hence there is potential for reporting bias.

The strength of the study, this study used a recall period of 2 weeks, which could minimize the effect of recall bias.

## Conclusion

Healthcare-seeking behavior for childhood illnesses had the potential to reduce childhood morbidity and mortality. However, caregiver’s healthcare-seeking behavior to child illness is low in this study setting. Three in ten caregivers were not sought healthcare for their child’s illness.

Caregivers’ educational level, knowledge of childhood illnesses, perceived severity of illness, and cough and diarrhea as main symptoms of illness were predictors of healthcare-seeking behavior for child illness. Therefore, to improve the healthcare-seeking behavior of child caregivers’ programs need to consider activities that enhance the knowledge of childhood illness and danger signs at the community level and need to give additional attention to illiterate caregivers.

## Data Availability

Data will be available upon reasonable request from the corresponding author.

## Abbreviations

ARI: Acute respiratory infection
CSA: Central statistics agency
EDHS: Ethiopian demographic and health survey
HEP: Health extension program
HF: Health facility
IMNCI: Integrated management of childhood illnesses
MDG: Millennium development goal
ORS: Oral rehydration salt
PPS: Probability proportional to size
UNICEF: United Nations international child’s fund
WHO: World health Organization
